# Rational and irrational vaccine hesitancy

**DOI:** 10.1186/s13584-023-00560-1

**Published:** 2023-03-28

**Authors:** Manfred S. Green

**Affiliations:** School of Public Health, University of Haifa, 199 Abba Khoushy, Mount Carmel, 3498838 Haifa, Israel

**Keywords:** Vaccine hesitancy, Rational, Irrational, COVID-19

## Abstract

In the paper published recently in this journal, Kumar et al. explained why the key to improved COVID-19 vaccine uptake is to understand societal reactions leading to vaccine hesitancy. They conclude that communications strategies should be tailored to the different phases of vaccine hesitancy. However, within the theoretical framework provided in their paper, vaccine hesitancy should be recognized as having both rational and irrational components. Rational vaccine hesitancy is a natural result of the inherent uncertainties in the potential impact of vaccines in controlling the pandemic. In general, irrational hesitancy is based on baseless information obtained from hearsay and deliberately false information. Risk communication should address both with transparent, evidence-based information. Rational concerns can be allayed by sharing the process in which dilemmas and uncertainties are dealt with by the health authorities. Messages on irrational concerns need to address head on the sources spreading unscientific and unsound information. In both cases, there is a need to develop risk communication that restores trust in the health authorities.

## Background

Vaccine hesitancy remains a major threat to the uptake of effective vaccines, increasing the risks of serious vaccine-preventable diseases [1, 2]. COVID-19 vaccines appear to have engendered even greater hesitancy than has been encountered for other vaccines [3–7], posing a major challenge to the control of the current pandemic. Kumar et al. [8] describe how vaccine hesitancy has changed during the COVID-19 pandemic and varies according to the individual, family and society and is not uniform for all vaccines. For example, COVID-19 vaccine hesitancy is more common among women [9] and among young people [10, 11]. Vaccine hesitancy has been encountered in both low and high income countries [12–15] and must be considered in the context of the availability and accessibility of the vaccines. As of October 2022, over 70% of people in high-income countries had received at least one dose of COVID-19 vaccine compared with only 25% in low-income countries [16].

Kumar et al. [8] have provided a theoretical framework for understanding vaccine hesitancy by expanding the classical three to five Cs of vaccine hesitancy, by adding calculation and collective responsibility to complacency, convenience and confidence [17]. However, within this framework, vaccine hesitancy can be both rational and irrational. Whereas rational vaccine hesitancy is based on reasonable doubts about the vaccine, irrational beliefs tend to be “illogical, incorrect or distorted ideas that are firmly held by a person despite there being clear, objective and contradictory evidence to show otherwise” [18]. Rational hesitancy includes the perception that the disease may be less severe than reported by the authorities. Other rational arguments are that the efficacy of the vaccines may be lower than published and that there may be rare adverse effects that have not yet been detected. Irrational messages that have been promoted include the concern that the vaccine may contain a computer chip to access your private personal information or has long-term effects on fertility. It is my contention that the approaches used to counter vaccine hesitancy must allow for the possibility of both rational and irrational views.

## The phases of vaccine hesitancy

The rational and irrational components of vaccine hesitancy can be identified in the different phases of vaccine hesitancy described by Kumar et al. [8]. In the early stages of an epidemic or pandemic, the public is expected to trust a new vaccine with limited tools for assessing the efficacy of the vaccines and the potential for adverse effects. This is particularly problematic when reports in the media cast doubt on the efficacy of the vaccines and magnify reports on side-effects. Resistance may be rational if the information on the incidence and severity of the disease is either lacking or unclear. Weak messages on the importance of vaccination for protection against the disease can strengthen the irrational components of vaccine hesitancy.

## The social media and vaccine hesitancy

The social media have become a major source of information on vaccines for the general public [3, 19–21] and can impact on both rational and irrational vaccine hesitancy. This can be particularly damaging when baseless or even deliberately false information is published without balanced factual information. For example, there may be strong messages on the media proposing that the pharmaceutical industry is withholding information on the safety of the vaccines that may adversely affect their sales [22]. In practice, regulatory agencies such as the FDA intensively scrutinize the safety and efficacy data from the clinical trials and they are joined by national health authorities who monitor post-marketing safety and effectiveness data. An irrational conspiracy theory is that the vaccines are being promoted to prevent the use of much cheaper (although unproven) medications such as Ivermectin [23, 24] and hydroxychloroquine [25] for treatment of the disease.

Irrational hesitancy can be reinforced by messages which are often overly simplistic and based on superficial interpretations of the available information. For example, short messages that the vaccine is not useful for children are readily accepted as being based on facts without providing more detailed evidence. Unfounded personal views published on the social media are widely read before they are confirmed or refuted. Even after countering false claims, they remain in the memory of the viewers. This can stimulate rational vaccine hesitancy or reinforce the fears of those who have irrational reasons for refusing vaccination.

An interesting phenomenon encountered during the COVID-19 pandemic is the emergence of social media groups claiming that the authorities have created unnecessary panic by exaggerated claims of the dangers of the disease to justify the introduction of preventive measures. Some groups were composed of people with impressive titles and degrees, although not necessarily from the field of infectious diseases and vaccines. This can be particularly confusing since the public cannot easily distinguish between those who are true experts or come from a quite different area of expertise. While open discussion of the dilemmas facing the decision-makers is to be encouraged, those with professional qualifications from an unrelated field should take particular care when criticizing the decisions of the authorities. Such criticism may unjustly erode the trust in the public health authorities.

## Examples of rational vaccine hesitancy

The dilemmas associated with choosing whether or not to be vaccinated can be compared to approach-avoidance conflict. Even if the vaccine appears to be effective, there are many rational factors which may lead some to question the safety of the vaccine [3, 18]. The rapid development of the COVID-19 mRNA vaccines produced the rational concern that this may not allow for adequate testing [26]. What was not always appreciated was that in unusual public health emergencies, such as the COVID-19 pandemic, enormous additional resources are mobilized. A lack of understanding of the process of the introduction of new vaccines under emergency use approval (EUA) [27], can create a perfectly rational concern about the safety and efficacy of the vaccine. The concept of EUA was often misinterpreted and misreported to mean that the vaccine was authorized as a vaccine still in the clinical trial phase. In fact the regulatory authorities issue EUA of vaccines if their effectiveness in preventing, diagnosing, or treating a disease, outweighs the known and potential risks of the vaccine [28].

The COVID vaccines authorized by the FDA under EUA went through the same standard phases of vaccine testing including adequately powered randomized, double-blind controlled clinical trials, in the general population. For full authorization, the FDA usually requires data on the participants in the trial for a median of six months follow-up [28]. In a public health emergency, this period of time can be shortened to a median of two months. In addition, the FDA expects that an EUA request will include a phase 3 safety database of well over 3,000 vaccine recipients, who have been followed for serious adverse events for at least one month following the full vaccination regimen. Since in general, both mild and severe adverse effects of vaccines are usually seen within the first month of follow-up, two months was considered a more than adequate time for assessing the efficacy and safety of the vaccines. In practice, the COVID mRNA vaccines soon exceeded the median period of six months and have been given full FDA approval [28]. In accordance with standard practice for vaccines, they are constantly monitored for safety in the post-authorization phase.

Another rational factor affecting vaccine hesitancy may be the lack of a clear national immunization policy. The continuing evolution of the COVID-19 pandemic has created difficulties in defining such a policy. This is largely due to the gradual accumulation of evidence on the duration of immunity produced by the vaccines and the need for further doses. In addition, new strains of the virus continue to emerge and this resulted in new formulations of the vaccine, creating confusion about the efficacy of the earlier vaccines against the new strains. This can add to public uncertainty about the importance of additional doses and the efficacy of the vaccines against new strains, which in turn may lead to rational vaccine hesitancy regarding the booster doses.

Rational vaccine hesitancy can also occur with vaccines that have known rare side-effects accompanied by a low perceived risk of the disease. This may be associated with an unrealistic public demand for a no-risk scenario. For example, the mRNA vaccines are associated with a small risk of myocarditis, mainly in young males, which is usually self-limiting [29]. The response to this concern should be to provide simplified rational information on the efficacy of the vaccine and relatively low risk of side-effects compared with the risk and potential adverse effects of the disease (which itself has a higher risk of myocarditis). In any event, transparent information on side-effects from the vaccine compared with the benefits is critical for conveying messages to the public. In this context it is important for the authorities to share dilemmas with the public and clarify the issues of individual protection and population control [3].

## Example of irrational vaccine hesitancy

Interestingly, the use of COVID-19 vaccines became a major political issue in the United States [30] and elsewhere [31], where irrational arguments related to the COVID-19 vaccines, such as conspiracy theories, abounded [24]. This clearly impacted on vaccine hesitancy. There is evidence that in states that have a majority of Republicans, there was a lower uptake of the COVID-19 vaccines and higher disease rates and deaths [32]. In a particular instance, in the state of Florida, a non-peer reviewed report of a study from the health commissioner’s office indicated excess all-cause and CVD mortality following mRNA vaccination in selected sub-group, which led to a precipitous decision to withdraw approval of mRNA vaccines in males aged 18–39 [33]. This was done despite the fact that there appeared to be important methodological issues with the study and the results contradicted findings in other studies. Such publications can create irrational vaccine hesitancy and the authorities should rapidly provide the public with professional analyses of the study and its limitations.

Rational COVID-19 vaccine hesitancy should be cleared distinguished from the ideological irrational views of those in the anti-vaccine movements [24, 34–40]. The followers often have deep seated beliefs that some or all vaccines are inherently bad, dangerous and or useless [41–43]. They represent a different category from the larger group of vaccine hesitant people. They are frequently much more influenced by the social media [44], by high profile personalities and by publications of questionable data [23]. They are likely to quote data selectively to support their preconceived ideas, rejecting studies that are not concordant with their beliefs. They tend to be very consistent in their objections to the vaccine, regardless of what new information is presented. They represent the extreme spectrum of irrational vaccine hesitancy and require a different approach to that needed for rational vaccine hesitancy.

## Dealing with rational and irrational vaccine hesitancy

Within the theoretical framework provided by Kumar et al. [8], both rational and irrational aspects of vaccine hesitancy should be clearly recognized and should not be ignored in public messages. Rational vaccine hesitancy does not imply that the hesitancy is justified. Rather it is likely that concerns about the vaccine can be addressed with transparent and evidence-based information while avoiding information overload. This could be done in the framework of general health promotion. As Kumar et al. [8] have stated, the key to improved vaccine uptake is to better understand vaccine hesitancy and develop better communication strategies supported by high quality data. They argue that there is a need for different strategies of communication to deal with the various nuances of all phases. They stress that to address of vaccine hesitancy, an understanding of the societal reactions leading to various phases of vaccine hesitancy is of utmost importance.

Since messages on the social media are particularly problematic when there is a lack of authoritative data, poor quality of available data and deliberate misinterpretation of the data. The public should be provided with the necessary tools to judge both the irrational and rational information on the vaccine.

## Information overload with the COVID vaccines

Paradoxically, while transparency is essential for combatting vaccine hesitancy, information overload can have a negative impact. One of the unique characteristics of the rollout of the COVID vaccines was the extraordinary amount of information that rapidly became available to the professional and lay public. Generally, the information on the efficacy and safety of new vaccines is released gradually over time and is mainly covered by the professional literature. Once the clinical trials have been completed and the new vaccines are introduced, there is a considerable lag time before effectiveness data based on post-marketing studies is published.

In the case of the COVID pandemic, post-marketing effectiveness data on the vaccines were made available very soon after the vaccines were introduced for general use. This was due to the unique situation where a large number of people were immunized during a short period of time during a period of high incidence and mortality rates from the disease. This was combined with the recent evolution of large and comprehensive administrative electronic databases containing personal information on immunization status and infections from COVID-19. The medical journals were overwhelmed with papers reporting studies based on these databases. In addition, the journals made a special effort to ensure rapid turnaround from submission to review to publication. The published studies were immediately reported in the lay press and made available to the general public. The public was then exposed to large volumes of information on the safety and efficacy of the vaccines in different subgroups, over different periods of follow-up, different vaccination formulation, efficacy against changing strains of the coronavirus and side-effects of the vaccine. This phenomenon was completely new and resulted in the paradox of information overload both for professionals and the public.

In practice, it was inevitable that there would be studies with conflicting or unclear results which would generate mixed and confusing messages. The lay media cannot adequately cover the complexities of the studies and their limitations. For example the public was expected to understand subtle differences between issues such as whether the vaccine completely prevents infection or only mild or severe disease. Questions were raised about whether vaccines prevent those immunized from spreading the virus. This created conditions of uncertainty which understandably could impact on rational vaccine hesitancy and on the public trust of the decisions made by the authorities.

## Conclusions

The COVID-19 pandemic has highlighted the need for a better understanding of the rational and irrational aspects of vaccine hesitancy. Both rational and irrational vaccine hesitancy may be manifestations of legitimate concerns and should be addressed with concise, evidence-based and timely information. A lack of transparency by the health authorities may contribute to the perception that there are no facts, only opinions [45]. Rational concerns can be allayed by sharing the uncertainties resulting from gaps in the evidence together with explanations how they are accounted for in the decision-making process. Addressing irrational vaccine hesitancy requires a somewhat different approach. It is important to show empathy for those holding irrational views and not to deny their right to hold these views. Efforts should be made to identify the sources of information that are reinforcing irrational concerns and address them with rational evidence. Attempts should be made to guide the discussion to actual known facts and look for possible agreement on at least some of the issues. Finally, vaccine hesitancy should be viewed in the framework of general health promotion [46–48] and lessons can be learned from approaches to other health-related behaviors. Focused research should be encouraged to determine the best practices for promoting vaccine uptake among those with both rational and irrational concerns.

## Data Availability

Not relevant.

## Abbreviations

FDA: United States Food and Drug Administration
EUA: Emergency Use Authorization
